# Age-related variations of visuo-motor adaptation beyond explicit knowledge

**DOI:** 10.3389/fnagi.2014.00152

**Published:** 2014-07-04

**Authors:** Herbert Heuer, Mathias Hegele

**Affiliations:** ^1^Leibniz Research Centre for Working Environment and Human Factors, DortmundGermany; ^2^Neuromotor Behavior Laboratory, Department of Sport Science, Justus-Liebig-University GiessenGiessen, Germany

**Keywords:** visuo-motor rotation, reinforcement learning, model-based learning, explicit knowledge, after-effect

## Abstract

Visuo-motor adaptation suffers at older working age. The age-related decline of behavioral adjustments is accompanied by reduced explicit knowledge of the visuo-motor transformation. It disappears when explicit knowledge is kept constant across the age range, except for particularly high levels of explicit knowledge. According to these findings, at older adult age both the acquisition of explicit knowledge and its application for strategic corrections become poorer. Recently it has been posited that visuo-motor adaptation can involve model-free reinforcement mechanisms of learning in addition to model-based mechanisms. We tested whether age-related declines of reinforcement learning can also contribute to the age-related changes of visuo-motor adaptation. Therefore we enhanced the contribution of reinforcement learning to visuo-motor adaptation by way of introducing salient markers of success and failure during practice. With such modified practice conditions, there were residual age-related variations of behavioral adjustments at all levels of explicit knowledge, even when explicit knowledge was absent. The residual age-related variations were observed for practiced target directions only, but not for new target directions. These findings are consistent with an age-related decline of model-free reinforcement learning as a third factor in the age-related decline of visuo-motor adaptation. Under practice conditions, which spur model-free reward-based learning, this factor adds to the decrements of the acquisition of explicit knowledge and its use for strategic corrections.

## INTRODUCTION

Since the seminal study of Cunningham (1989), adaptation to visuo-motor rotations has become a popular paradigm for the study of the plasticity of the human brain. Typically, participants perform aimed movements to control the position of a cursor on a computer monitor. The direction of cursor motion is rotated relative to the direction of hand movement. In the course of practice, participants gradually reduce reaching errors and return performance to pre-perturbation (without visuo-motor rotation) levels. With this and other types of visuo-motor transformations, age-related variations of adaptation have been shown (e.g., McNay and Willingham, 1998; Buch et al., 2003; Bock, 2005; Bock and Girgenrath, 2006; Heuer and Hegele, 2008, 2009; Hegele and Heuer, 2010a). However, adaptation to a visuo-motor transformation is not a unitary process, but embraces different processes which comprise distinct components that contribute to the total outcome (e.g., Saijo and Gomi, 2010). In the present study we test the sensitivity of certain components of adaptation to aging.

An important distinction is the one between implicit and explicit components of visuo-motor adaptation (Mazzoni and Krakauer, 2006; Sülzenbrück and Heuer, 2009; Hegele and Heuer, 2010a,b,c; Taylor et al., 2010, 2014; Heuer et al., 2011; Taylor and Ivry, 2011). Implicit components of adaptation are not subject to conscious awareness. Explicit components, in contrast, are intentional movement corrections that are based on explicit knowledge of the transformation. With respect to measuring implicit and explicit components, implicit components are generally assessed by after-effects in the absence of the transformation. Explicit knowledge can be assessed by means of perceptual judgements on movement parameters which are thought to be adequate for correct movements in the presence of the visuo-motor transformation.

Explicit and implicit components of adaptation to visuo-motor rotations differ in a variety of ways. For example, explicit components generalize across all target directions in the workspace, whereas implicit components are restricted to the practiced direction and a limited range around it (Heuer and Hegele, 2008). Most important for the present purpose, explicit components are reduced across the adult age range, whereas implicit components remain stable. These age-related changes have been shown in a number of independent studies and with different experimental protocols (e.g., McNay and Willingham, 1998; Buch et al., 2003; Bock, 2005; Bock and Girgenrath, 2006; Heuer and Hegele, 2008; Hegele and Heuer, 2010a,b), including the demonstration of absent age-related changes with small rotations (e.g., Heuer and Hegele, 2008, Exp. 2) or their slow introduction analogous to “prismatic shaping” (Dewar, 1971) which does not give rise to explicit knowledge (e.g., Buch et al., 2003; Cressman et al., 2010). The age-related changes in the explicit component have been hypothesized to be related to structural changes in particular of the frontal lobes (e.g., Heuer and Hegele, 2008) and to functional changes such as increased neural noise and neural de-differentiation (e.g., Rand et al., 2013). Such changes should result in a reduced sensitivity for the difference between the directions of hand movements and cursor motions, which has been reported by Rand et al. (2013). Therefore older adults should perceive this difference as smaller than young adults or even not at all.

However, age-related variations of explicit knowledge are only partly responsible for the age-related differences in overall adaptation. Residual age-related variations at same levels of explicit knowledge indicate the effects of additional factors. For example, Hegele and Heuer (2013) observed stronger behavioral adjustments in young than in older adults with high explicit knowledge, whereas no such difference was seen between young and older adults with poor explicit knowledge. This finding suggests that not only the acquisition of explicit knowledge suffers at older age, but also its use for behavioral adjustments. However, age-related variations of behavioral adjustments across all levels of explicit knowledge, and thus also in its absence, have not yet been found. Here we test whether a certain modification of the practice conditions gives rise to such differences between young and older adults. This test was motivated by the following considerations (1) on a role of reinforcement learning in visuo-motor adaptation and (2) on age-related declines of the dopaminergic neurotransmitter system that plays an essential role in reinforcement learning.

Implicit processes of visuo-motor adaptation have generally been conceptualized in terms of the acquisition of an internal model (cf. Shadmehr et al., 2010). In addition to such model-based learning, model-free reinforcement learning has recently been proposed also to contribute to visuo-motor adaptation (e.g., Huang et al., 2011; cf. Haith and Krakauer, 2013, for review). While both, model-free and model-based learning, are driven by prediction errors, those errors differ in terms of the informational content. Model-based learning is driven by sensory prediction errors that reflect the violation of expectations regarding affectively neutral sensory signals. In case of visuo-motor adaptation, its product is an internal representation of the transformation. Model-free reinforcement learning, in contrast, is driven by reward-prediction errors, which reflect the difference between an expected and the actual reward. Its product is an association between sensory stimuli and a particular movement (or set of movements) that maximizes future rewards (Sutton and Barto, 1998). In visuo-motor adaptation, motor adjustments based on model-free reinforcement learning should take the input-output relations of the transformation into account and thus imply learning of the transformation. Such learning, however, should be limited to the practiced type of movements and should not lead to the development of an internal model which could be applied to new types of movements (cf. Heuer, 1983, pp. 83–84; Bock, 2005; Bock and Girgenrath, 2006).

The different implicit-learning mechanisms involve different neural substrates (e.g., Doya, 2000). The sensory prediction error used to acquire a model of the novel visuo-motor transformation has been shown to correlate with characteristic changes in the BOLD signal in the intraparietal sulcus and the lateral prefrontal cortex (Gläscher et al., 2010). In addition, the cerebellum has been shown to be critically involved in the acquisition of (implicit) internal models of novel visuo-motor transformations (Taylor et al., 2010; Imamizu and Kawato, 2012; Schlerf et al., 2012). Reward prediction errors used by model-free learning, in contrast, are correlated predominantly with activity of subcortical structures such as that of the midbrain dopaminergic system (Schultz et al., 1997; Schultz, 1998, 2007; Bayer and Glimcher, 2005). The dopaminergic system is among those neurotransmitter systems which are known for age-related declines and associated behavioral changes (Volkow et al., 1998), among them changes in reward-based learning (Marschner et al., 2005; Dreher et al., 2008). Thus, to the extent that model-free reinforcement learning contributes to adaptation to a novel visuo-motor transformation, age-related changes beyond those related to explicit knowledge would be expected.

In previous studies of age-related variations of visuo-motor adaptation, practice was typically with rapid uncorrected out-and-back movements with rotated visual feedback (e.g., Bock, 2005) or accurate movements under closed-loop control that reached their targets without severe time constraints (e.g., Heuer and Hegele, 2008). With tasks such as these there are no clear categorical indicators of success or failure. With rapid uncorrected out-and-back movements there are graded deviations of the reversal positions from the targets, and with accurate movements under closed-loop control there are graded deviations from straight paths and graded variations of movement durations before the targets are reached. Without categorical indicators of success and failure in such tasks, reward-based reinforcement learning should be of only little importance, if at all. In the present study we introduced a clear categorical marker of success or failure during practice, namely a hit or miss in a virtual putting task. Such a marker should serve to enhance the contribution of reward-based learning (cf. Izawa and Shadmehr, 2011). If the modified practice conditions indeed facilitated model-free reinforcement learning based on reward prediction errors, they should result in an age-related decline of visuo-motor adaptation at all levels of explicit knowledge, even in its absence.

## MATERIALS AND METHODS

### PARTICIPANTS

Two groups of young and older participants served in the experiment. All participants had given written informed consent, had normal color vision according to the Ishihara test, and were self-declared right-handers. The younger participants, 9 male and 10 female, were 18–31 years old (mean: 23.5 years, SD: 3.2 years). The older participants, 8 male and 10 female, were 47–67 years old (mean: 57.2 years, SD: 5.6 years). The data of three additional older participants were not included in the analyses because under at least one of the conditions tested they produced quite irregular movements (e.g., systematic movements in the direction opposite to target direction or spiral movement paths). The experiment was done in accordance with the ethical standards laid down in the Declaration of Helsinki.

The two groups of participants were compared on the Digit Symbol Test of the German version of the Wechsler Adult Intelligence Scale (Tewes, 1991) and on a Vocabulary test, the MWT-B (Lehrl, 2005). Performance in these tests depends in characteristic ways on age. Deviations from these characteristic differences can serve as indicators of cognitive differences between the two age groups that are not related to age. The Digit Symbol Test assesses perceptuo-motor processing speed. For this test an age-related decline of performance is typical, which was also found for the two age groups of this experiment. The means of the young and older participants were 67.7 and 48.9, respectively, *t*(35) = 5.0, *p* < 0.01. The Vocabulary test is a test of culturally mediated knowledge. Performance in this type of test is typically robust across the lifespan, sometimes even better in older than in young adults. The mean scores of the current groups of young and older participants were 27.3 and 32.0, respectively, *t*(35) = 3.7, *p* < 0.01.

### APPARATUS

Participants sat on a chair and faced a 19″ LCD monitor (Iiyama ProLite E1902S) which was placed on a table at a distance of about 100 cm from their eyes. Their right index finger was strapped to a sled of 50 mm x 30 mm (height: 6 mm) which carried a vertically oriented sensor of a miniBIRD 800 system (Ascension Technology, Burlington, VT) directly above the participant’s finger nail. The sled slid with only little friction on the table surface. The position of the fingertip was recorded at 103.3 Hz (spatial resolution: 0.11 mm). An opaque cover 20 cm above the table surface prevented direct vision of the hand. Custom made software for the control of the experiment was written in MATLAB using the Psychtoolbox (Brainard, 1997; Pelli, 1997; Kleiner et al., 2007).

### TASKS

Participants practiced aimed movements in the context of a virtual putting task. Tests involved aimed movements without and with visual feedback (movement tests) as well as explicit judgements of presumably correct movement directions. All movements shared a visually presented start position in the center of the monitor, which was associated with a start position of the index finger on the table about 30-40 cm in front of the participant and about 15 cm to the right of the participant’s median plane. The color of the circle, which marked the start position on the monitor, served to cue the presence or absence of a visuo-motor rotation of 75° clockwise (-75°). A green color of the start circle cued the absence of the rotation, a red color its presence. Participants were instructed that hand and cursor moved in the same direction when the start circle was green, and that the direction of the cursor was rotated relative to the direction of the hand when the start circle was red, but nothing was said about either the polarity (clockwise or counterclockwise) or the magnitude of the rotation.

In the virtual putting task that was performed during practice, a “ball” was presented on the monitor at a distance of 30 mm from the start location and a “hole” at a distance of 120 mm. The ball and the hole were located along the same radial line emanating from the start location. Their directions could be 0° (to the right), 45°, 90° (forward on the table, upward on the monitor), 135° or 180°. The participants had to hit the ball with the cursor such that it reached the hole. The movement of the cursor from the start position to the ball had to be smooth and without interruption. When the ball was hit, the cursor disappeared from the monitor, and the ball moved in a direction determined by the location of impact at an initial velocity determined by the velocity of the cursor orthogonal to the surface of the ball. When the damped motion of the ball was long enough so that it reached the hole, a tone sounded and the trial was ended. When the ball did not reach the hole, its final position was shown for 0.2 s before the trial was ended. When the cursor missed the ball, the trial ended as soon as cursor velocity approached zero.

In the movement tests, targets were presented at distances of 30 or 120 mm from the start location at directions of 0°, 45°, 90°, 135°, 180°, 225°, 270°, or 315°. The first five of these directions were also used during practice, but the last three were not. The target amplitudes corresponded to the distances of the ball and the hole from the start position in the practice task. Movements were performed without and with visual feedback of the cursor position in different tests.

In each trial of the explicit-judgment test, the start location and a target at a distance of 120 mm were presented. All eight target directions were used. A line was presented with its one end fixed in the start location. Initially it pointed into a randomly chosen direction. The participant instructed the experimenter to rotate the line clockwise or counter-clockwise until it pointed in a direction that corresponded to the direction of hand movement appropriate for a cursor on the monitor to reach the target position. The start circle was either red or green to cue the presence or absence of the visuo-motor rotation.

### DESIGN AND PROCEDURE

An overview of the various phases of the experiment is given in **Table 1**. The experiment started with a block of 40 familiarization trials with the putting task in the absence of the visuo-motor rotation (green start circle). Thereafter pre-tests, practice, and post-tests followed.

**Table 1:** Overview of experimental phases.

Phase		Type of block	Task	Visual feedback	Visuo-motor rotation	Number of trials	Number of repetitions
1	Familiarization		Putting	Yes	No	40	1
2	Pre-tests	Maintenance	Putting	Yes	No	5	3
		Open-loop test	Aiming 30 mm	No	No	8	
		Maintenance	Putting	YesNo	No	5	3
		Open-loop test	Aiming 120 mm	No	No	8	
		Maintenance	Putting	Yes	No	5	2
		Explicit test	Judgment	–	No	8	
3	Practice		Putting	Yes	Yes	40	8
4	Post-tests	Maintenance	Putting	Yes	Yes	5	3
		Open-loop test	Aiming 30 mm	No	Yes	8	
		Maintenance	Putting	Yes	Yes	5	3
		Open-loop test	Aiming 120 mm	No	Yes	8	
		Maintenance	Putting	Yes	Yes	5	3
		Open-loop test	Aiming 30 mm	No	No	8	
		Maintenance	Putting	Yes	Yes	5	3
		Open-loop test	Aiming 120 mm	No	No	8	
		Maintenance	Putting	Yes	Yes	5	2
		Explicit test	Judgment	–	Yes	8	
		Cosed-loop test	Aiming 120 mm	Yes	Yes	40	1

Pre-tests were without visuo-motor rotation; the start circle was green in all trials. There were two visual open-loop tests with short (30 mm) and long (120 mm) target amplitudes. In the open-loop tests, the cursor became invisible after the start of each movement and did not re-appear until the start position was approached again for the next trial. In addition, there was an explicit test. Each of the two movement tests consisted of 3 blocks of 8 trials each, one trial for each of the eight target directions. Each of these three test blocks was preceded by a maintenance block of five trials with the putting task, in which each of the five target directions was presented once. The explicit test consisted of only two rather than three maintenance-test cycles.

Practice consisted of eight blocks of 40 trials each. There was a pause of at least 2 min after the seventh block to allow recovery from eventual fatigue before the last block of practice and the subsequent tests. In each trial the putting task was performed with a visuo-motor rotation of -75° (clockwise). The presence of the rotation was cued by the red color of the start circle. In the 40 trials of each block eight random permutations of the five target directions were presented (without repetitions of target directions).

Post-tests consisted of visual open-loop tests with the presence of the visuo-motor rotation cued, both with short and long target amplitudes, and with the absence of the rotation cued, again both with short and long target amplitudes. The open-loop tests were followed by an explicit test in which the presence of the rotation was cued. Open-loop tests consisted of three maintenance-test cycles as the pre-tests, and the explicit test of two such cycles. Maintenance trials were identical to practice trials. Finally there was a block of 40 visual closed-loop trials in which aimed movements to the long-amplitude targets were performed with the visuo-motor rotation being in effect. Each of the eight target directions was presented five times in this block. We added this final test in which visual feedback was continuously presented during each movement to determine whether the age-related variations, as assessed in the absence of visual feedback, are also relevant for performance when closed-loop control is possible. Closed-loop control seems to be more typical than open-loop control for tasks of everyday life in which visuo-motor transformations are present, e.g., in using a rake, a shovel or some other conventional tool. In addition, modern technologies create novel and challenging tool-use tasks in fields such as laparoscopic surgery and micro manipulation which are generally performed under visual closed-loop conditions.

All movement trials began with the presentation of a red or green outline circle of 9.6 mm diameter which marked the cursor start position on the monitor and cued the presence or absence of the visuo-motor rotation in the forthcoming trial. Arrows were presented at the left, right, lower or upper edge of the monitor, one or two arrows at the same time, which pointed to the center and thereby guided the participants to the start location. At a distance of less than 10 mm from the center of the start circle, the cursor became visible to assist in reaching the start position accurately. There was no visuo-motor rotation during this homing-in. The cursor was a cyan filled circle of 6 mm diameter. When the cursor was within a tolerance of 2 mm around the start position for 0.5 s, the start circle was filled, and after a waiting time of 1.4 s a target was presented.

For the practice task, the ball and the hole were presented at distances of 30 and 120 mm, respectively, from the start position. The ball was a white filled circle of 12 mm diameter, the hole a gray filled circle of 30 mm diameter. When the cursor missed the ball, the trial was finished as soon as the distance between two successive samples of the hand position was less than 0.16 mm. The trial was also finished when this criterion was satisfied while the cursor was on the way to the ball. This measure served to ensure smooth movements and to prevent a strategy of moving the cursor quite close to the ball and then “kicking” it. This criterion for the end of the trial also prevented movement corrections once the ball had been missed.

When the cursor contacted the ball, that is, when the distance between the centers of the cursor and the ball was less than the sum of their radii, the ball was set into motion, and the cursor disappeared from the monitor. The direction of motion was determined by the location of impact. More precisely, it was in the direction of the vector from the center of the cursor to the center of the ball. The initial velocity of the ball was 0.5 times the velocity of the cursor in that direction. To simulate friction of the ball, velocity in time step n + 1 was computed as v_n+1_ = 0.99v_n_. When the ball reached the hole, which required a correct location of impact and a cursor velocity at impact above 20 mm/s, a brief tone sounded and the trial was ended. When the ball missed the hole, it stopped when the distance between successive samples of the ball position became less than 0.16 mm or when it reached the edge of the monitor; 0.2 s later the trial was ended.

In aimed-movement trials, a white or gray filled circle of 7 mm diameter was presented as the target. The circle was white when the target amplitude was 30 mm, and it was gray when the target amplitude was 120 mm, corresponding to the colors of the ball and the hole in the practice task. For movements without visual feedback, the cursor disappeared when the target was presented. The trial ended when the distance between successive samples of the hand position was less than 0.16 mm for 500 ms, provided the cursor had left the tolerance around the start position. For movements with visual feedback the cursor remained visible, and the trial ended when the cursor was on the target for 0.5 s, with the tolerance around the target being the sum of the radii of target and cursor.

For explicit-judgment trials, a red or green start circle was presented together with a target in 120 mm distance from the start position and a line of 4 pixels width. The one end of the line was fixed in the start position. The length of the line corresponded to the start-target distance. Its orientation was changed by the experimenter according to the verbal instructions given by the participant. The participant continued to give verbal instructions until he or she judged the direction, indicated by the line, as corresponding to the direction of the hand movement appropriate for the (invisible) cursor on the monitor to reach the target.

### DATA ANALYSIS

Data were analyzed by means of custom made MATLAB programs. The time series of finger positions were low-pass filtered (fourth-order Butterworth, 10 Hz, dual pass) and differentiated (two-point central difference algorithm). The velocity signals were again low-pass filtered. Beginning and end of each movement were determined from tangential velocity of the hand. Beginning at peak velocity, in a backward and a forward search those samples were defined as start and end, respectively, at which tangential velocity became smaller than 5 mm/s and remained so for the next 250 ms. In practice trials the velocity criterion for the end of the movements was applied to misses only; for hits the movements ended at the moment of contact with the ball.

In total there were 654 movement trials for each participant. The initial 40 familiarization trials and the 110 maintenance trials were neglected. The remaining 504 trials were screened for the following irregularities: movement time was shorter than 150 ms (putting task) or 200 ms (aimed movements); movement time was longer than 5000 ms; path length was longer than 5 times the distance from initial to final position; initial position deviated more than 12 mm from the start location (this could happen when the main movement was preceded by a short initial movement); for aimed movements with visual feedback the amplitude error was larger than 10 mm or the direction error larger than 10°. In total 95.7% of all trials were included in the analyses for the young participants and 96.3% for the older participants. For all participants the proportion of discarded trials was less than 10% with the exception of two participants with 10 and 14%, respectively.

Both for the putting task and the aimed-movement task a number of variables were computed for the individual movements. For the putting task, these were hit versus miss, initial direction error, and movement time. (Hit versus miss refers to the cursor hitting the ball, as this turned out to be quite difficult with the visuo-motor rotation being in effect.) For the aimed-movement task, the dichotomous measure of hit versus miss was replaced by the final direction error. Final direction was measured as the direction of the vector from the initial to the final position of the movement, initial direction as the direction of the vector from the initial position of the movement to the position 200 ms later. The respective direction errors were the deviations of final and initial movement direction from the direction of the target. Counter-clockwise direction errors were positive, clockwise errors were negative. For each block of practice and each type of test, means of the dependent variables were computed across trials with the same target direction (for hit versus miss the proportion of hits was computed, and mean movement times in practice trials were computed separately for hits and misses). For each practice block these means were averaged, and for each test separate means were computed for the five practiced target directions and the three new target directions that were not used during practice.

From the final direction errors of the visual open-loop movements adaptive shifts and after-effects were computed. Adaptive shifts are the differences between the directions of hand movements in the post-tests in which the presence of the visuo-motor rotation is cued and in the pre-tests. Adaptive shifts of zero indicate no adaptation at all, whereas adaptive shifts of 75° indicate full adaptation which compensates the visuo-motor rotation of -75°. After-effects are the differences between the directions of hand movements in the post-tests, in which the absence of the visuo-motor rotation is cued, and in the pre-tests. They can be conceived as residual adaptive shifts that remain in spite of the knowledge that the visuo-motor rotation is not present. After-effects are thought to reflect primarily implicit components of adaptation, whereas adaptive shifts reflect explicit components in addition.

For the explicit judgements the changes from pre-test to post-test were determined in the same way as for the visual open-loop movements. The differences between the judged directions in the post-test and in the pre-test are called explicit shifts. Explicit shifts of zero indicate no explicit knowledge at all, and explicit shifts of 75° indicate perfect explicit knowledge. For the analysis of explicit shifts the data of two additional participants were discarded. In the post-test the one participant systematically judged the appropriate movements to be in the direction opposite the target. The other participant consistently judged the rotation to be in the wrong direction. Both participants did not produce correspondingly incorrect movements in the visual open-loop tests.

Parametric and non-parametric statistical tests as well as regression analyses were performed using STATISTICA. For the practice phase the proportion of hits, the initial direction error, and the movement time were subjected to two-way ANOVAs with the between-participant factor age group (young, older) and the within-participant factor block of trials (practice blocks 1-8); for movement time there was the additional factor hit versus miss. Degrees of freedom were Greenhouse-Geisser adjusted when appropriate, but we report the uncorrected degrees of freedom together with the Greenhouse-Geisser epsilon. Adaptive shifts, after-effects, and explicit shifts were subjected to three-way ANOVAs with the between-participant factor age group (young, older) and the within-participant factors target set (practiced, new) and target amplitude (short, long). For movement time in the visual open-loop tests only a single ANOVA was run with the type of test (pre-test, post-test with rotation, post-test without rotation) as a fourth factor. Individual differences in adaptive shifts, after-effects, and explicit shifts were analyzed with respect to eventual age-related variations of adaptive shifts and after-effects in the absence of age-related variations of explicit knowledge. For this purpose, residuals of the linear regressions of adaptive shifts and after-effects on explicit shifts and means of selected subgroups were compared between young and older adults using nonparametric tests. The final visual closed-loop test was analyzed by means of two-way ANOVAs with the between-participant factor age group and the within-participant factor target set.

## RESULTS

We report the findings for the practice phase first. Thereafter adaptive shifts, after-effects, and explicit shifts are reported together with movement times in the visual open-loop tests. Finally, we turn to the visual closed-loop performance as assessed at the end of the experiment.

### PRACTICE (PUTTING TASK)

**FIGURE 1:**
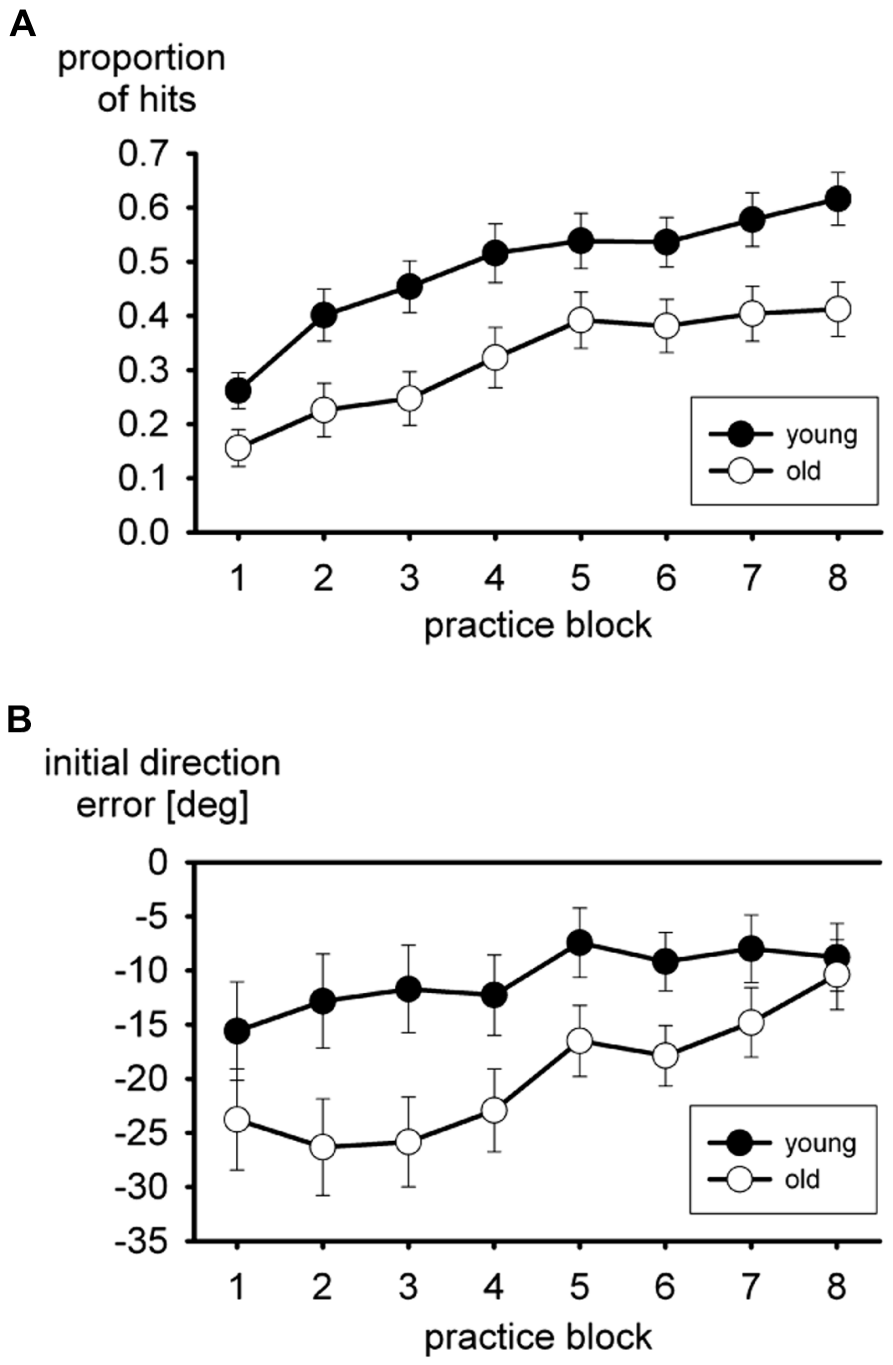
**Mean proportion of hits (A), and mean initial direction errors (B) during practice of the virtual putting task.** Error bars mark standard errors of the means.

Performance during practice of the putting task is shown in **Figure 1**. With a visuo-motor rotation of -75° it is fairly difficult to hit the ball with the cursor at all. The mean proportion of hits in the practice blocks is shown in **Figure 1A**. These are hits of the ball with the cursor, no matter whether the ball reached the hole. In both age groups the proportion increased in the course of practice, but a consistent advantage of the young group persisted. In the two-way ANOVA both the main effects of age group, *F*(1,35) = 7.8, *p* < 0.01, and block of trials, *F*(7,245) = 37.0, *p* < 0.01, ε = 0.65, were significant, but not the interaction, *F* < 1.

The mean initial direction error is shown in **Figure 1B**. Overall the initial direction error was negative, consistent with the visuo-motor rotation of -75°, more so in the older participants than in the young ones. It declined in the course of practice. The main effect of age group turned out to be significant, *F*(1,35) = 5.7, *p* < 0.05, in the two-way ANOVA, and the main effect of practice block as well, *F*(7,245) = 5.9, *p* < 0.01, ε = 0.51. The interaction did not approach statistical significance, *F*(7,245) = 1.4, *p* > 0.20, ε = 0.51, even though the difference between the two age groups tended to become smaller in the course of practice.

Some participants did not produce both hits and misses in each practice block. Therefore the analysis of movement time, which included the factor hit versus miss in addition to age group and practice block, was run with a reduced sample (15 of 19 young and 13 of 18 older participants). Older participants were slower than young participants, 457 vs. 376 ms, *F*(1,26) = 5.4, *p* < 0.05, and movement time declined in the course of practice, *F*(7,182) = 2.8, *p* < 0.05, ε = 0.53. Movement time of hits was shorter than movement time of misses, 251 vs. 502 ms for young participants and 367 vs. 546 ms for older participants. The main effect of hit versus miss was significant, *F*(1,26) = 49.3, *p* < 0.01, but not the interaction with age group,* F*(1,26) = 1.4, *p* > 0.20.

In an additional analysis we examined performance in the initial familiarization trials which differed from the practice trials only in that the visuo-motor rotation was absent. The proportion of hits was 0.90 and 0.82 for the young and older participants, respectively,* t*(35) = 1.90, *p* < 0.10, the initial direction error was 3.61 and 3.68°, *t*(35) = 0.04, *p* > 0.20. Movement time for hits was 303 and 351 ms, *t*(35) = 1.52, *p* < 0.20; misses were too rare for an analysis of their movement times. Thus, without the rotation there was a slight age-related variation of the same kind as in the practice trials (except for the initial direction errors). However, it failed to reach statistical significance. At least a major part of the age-related variation in the practiced putting task, therefore, resulted from the visuo-motor rotation.

### ADAPTIVE SHIFTS, AFTER-EFFECTS, AND EXPLICIT SHIFT

**FIGURE 2:**
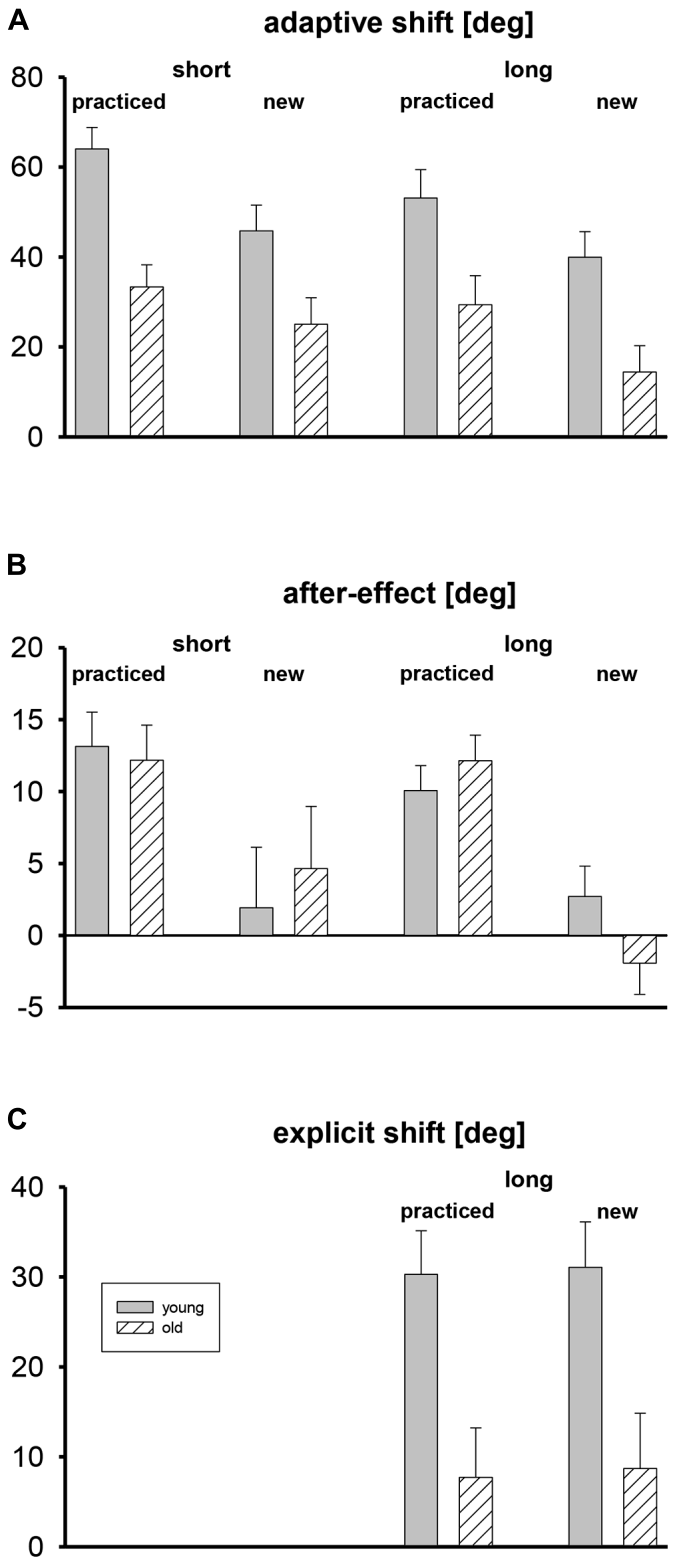
**Mean adaptive shifts (A), after-effects (B), and explicit shifts (C) in deg, shown separately for short and long target amplitudes, practiced and new target directions, and both age groups.** Error bars mark standard errors of the means.

Mean adaptive shifts, after-effects, and explicit shifts are shown in **Figure 2**, separately for the two age groups, tests with short and long target amplitudes, and the five practiced target directions and the three new target directions that were only used during the tests. For the assessment of explicit shifts only long target amplitudes were used.

Adaptive shifts were stronger in the young participants than in the older ones (**Figure 2A**). For the practiced target directions they were stronger than for the new ones, and for the short target amplitude they were slightly stronger than for the long one. The three-way ANOVA with the factors age group, target set (practiced, new), and target amplitude (short, long) revealed only significant main effects for age group, *F*(1,35) = 12.6, *p* < 0.01, for target set, *F*(1,35) = 22.3, *p* < 0.01, and for target amplitude, *F*(1,35) = 10.0, *p* < 0.01. For none of the eight means shown in **Figure 2A** did the 95% confidence interval include zero, that is, all adaptive shifts were reliably positive.

Mean after-effects are shown in **Figure 2B**. There were no differences between age groups, and for new targets after-effects were absent. The three-way ANOVA revealed only a significant main effect of target set, *F*(1,35) = 25.2, *p* < 0.01, but not of age group and target amplitude, and no significant interaction. The 95% confidence intervals of the mean after-effects for practiced target directions did never include zero, whereas the confidence intervals of the mean after-effects for new target directions always included zero.

In **Figure 2C** the mean explicit shifts are shown. They were clearly stronger in the young than in the older participants, and there was no difference between practiced and new target directions. A two-way ANOVA with the between-participant factor age group and the within-participant factor target set (practiced, new) revealed only a significant main effect of group, *F*(1,33) = 10.3, *p* < 0.01. The 95% confidence intervals included zero for the older participants, but not for the young ones.

Movement time was 552 ms overall in the visual open-loop pre-tests and the post-tests with and without the visuo-motor rotation. The four-way ANOVA with the factors age group, target set, target amplitude, and type of test revealed no significant effect involving the factor age group. Movement times were faster for short target amplitudes than for long ones, 516 vs. 589 ms, *F*(1,35) = 28.0, *p* < 0.01. The difference between movement times for long and short target amplitudes was larger in the pre-test than in the post-tests with and without rotation (110, 52, and 57 ms, respectively). The interaction of target amplitude and type of test was significant, *F*(2,70) = 5.9, *p* < 0.05, ε = 0.73. Finally, movement time was somewhat faster for the practiced target directions than for the new ones, 546 vs. 559 ms, *F*(1,35) = 7.1, *p* < 0.05.

### THE RELATION OF EXPLICIT KNOWLEDGE TO ADAPTIVE SHIFTS AND AFTER-EFFECTS

We analyzed the relations of the inter-individual variations of adaptive shifts and after-effects to the inter-individual variations of explicit knowledge according to the following rationale: Adaptive shifts, which are assessed in the cued presence of the transformation, should reflect model-free and model-based learning as well as strategic corrections based on explicit knowledge. For participants with same levels of explicit knowledge of the transformation, only model-free and model-based learning should contribute to age-related variations. In previous studies such variations have not been found, but for the present study with an enhanced contribution of model-free reinforcement learning they are expected. Thus, even after regression on explicit shifts the residual adaptive shifts of the two age groups should remain different. This age-related variation should be more pronounced for practiced target directions than for new ones because of the limited generalization of model-free learning.

After-effects, which are assessed in the cued absence of the transformation, should reflect implicit model-based learning. The color cue indicating the absence of the transformation should alleviate the need for any intentional strategic corrections. In addition it should serve as a discriminative stimulus for the non-applicability of acquired model-free associations between visual stimuli and motor responses. Thus, inter-individual variations of explicit knowledge should not covary with after-effects, and no age-related differences were expected.

For the analyses of the relation of explicit knowledge to adaptive shifts and after-effects, we averaged both the individual adaptive shifts and after-effects for short and long target amplitudes. The differences between target amplitudes were only small or absent, and the mean correlations between the individual measures were 0.80 and 0.71 for the adaptive shifts with practiced and new target directions, and 0.44 and 0.43 for the after-effects with practiced and new target directions (The mean correlations are inverse Fisher’s *z* transforms of the weighted means of Fisher’s *z* transforms of the correlations computed separately for each age group; they were all statistically significant.). Similarly, we averaged the explicit shifts for practiced and new target directions as the means were not different and the individual measures were highly correlated, with a mean correlation in the two age groups of 0.97. The averaging served to reduce the noise of the individual data.

**FIGURE 3:**
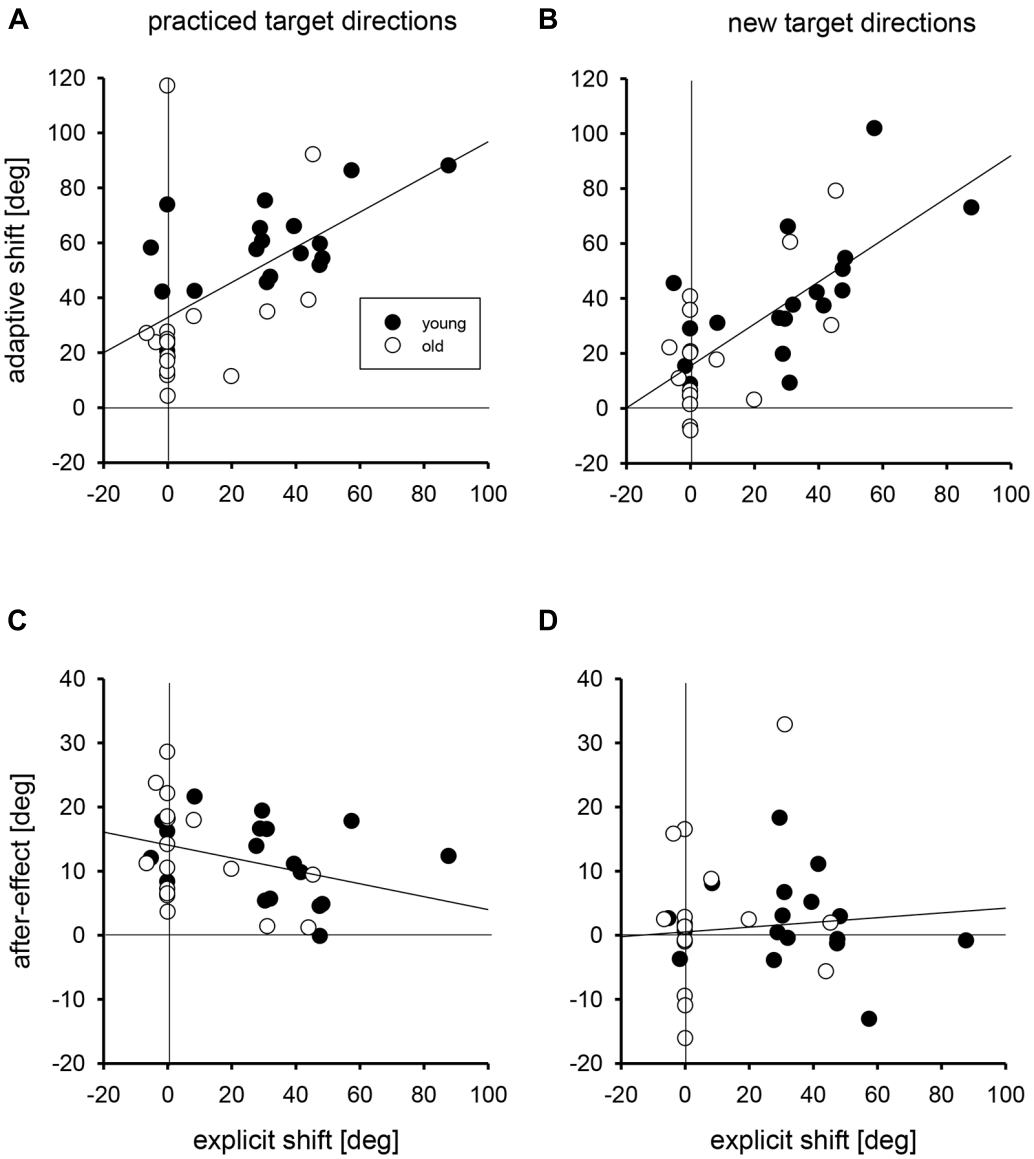
**The regressions of individual adaptive shifts for practiced (A) and new target directions (B) and of individual after-effects for practiced (C) and new target directions (D) on individual explicit shifts.** Filled circles mark the individual data points of young participants, open circles those of older participants. The zero points on the abscissae and ordinates are marked by vertical and horizontal lines, respectively.

In **Figure 3** the linear regressions of the individual adaptive shifts and after-effects with practiced and new targets directions on the individual explicit shifts are shown. For these regression analyses, young and older groups were collapsed, but the data points of the two groups are marked by different symbols in **Figure 3**. For adaptive shifts the correlations of 0.56 and 0.71 for practiced and new target directions were significant; the respective slopes of the linear regressions were 0.64 and 0.77 degree of adaptive shift per degree of explicit shift. For after-effects the correlations were -0.34 and 0.08, and only the first of these was significant. The respective slopes were -0.10 and 0.04 degree of after-effect per degree of explicit shift.

We performed two different analyses to determine whether the age-related variation of adaptive shifts could be attributed to the age-related variation of explicit knowledge, and we ran the same kind of analyses also for after-effects for which no age-related variation was observed. In the first analysis, we compared the residuals of adaptive shifts and after-effects after regression on explicit shifts between the two age groups. If there were no age-related variation beyond that in explicit knowledge, the mean residuals in both age groups should not deviate from each other (and from zero, which is the overall mean of the residuals). The mean residuals in the two age groups together with the results of Mann-Whitney *U*-tests are shown in **Table 2** (left half). Only for adaptive shifts with practiced target directions the residuals were larger in the group of young than in the group of older participants, that is, young participants had relatively stronger adaptive shifts than expected from their better explicit knowledge, whereas older participants had relatively weaker adaptive shifts than expected from their poorer explicit knowledge.

**Table 2:** Age-related variations of residuals of adaptive shifts (AS) and after-effects (AE) after regressions on explicit shifts (left half) and of AS and AE in subgroups without explicit knowledge (right half).

		Mean residuals of AS and AE			Mean AS and AE		
		Young	Old	*U* (18,17)	Young	Old	*U* (5,13)
AS	Practiced	5.87	-6.22	64, *p* < 0.01	47.3	28.0	12, *p* < 0.05
	New	1.54	-1.63	132, *p* > 0.10	25.8	13.0	17, *p* < 0.10
AE	Practiced	0.84	-0.89	124, *p* > 0.10	15.1	14.5	31, *p* > 0.10
	New	0.33	-0.35	145, *p* > 0.10	1.8	-1.8	27, *p* > 0.10

*Statistical comparisons are based on Mann–Whitney *U*-tests (one-tailed) with the test statistics *U* (*n*_young_, *n*_older_).*

The analysis of the residuals covers the whole range of explicit knowledge. In the second analysis we focussed on the 5 young and the 13 older participants without explicit knowledge (explicit shifts less than 10°). The mean adaptive shifts and after-effects of these subgroups are also shown in **Table 2** (right half) together with the results of Mann-Whitney *U*-tests. Again there was an age-related variation of adaptive shifts with practiced target directions – younger participants without explicit knowledge had larger adaptive shifts than older participants without explicit knowledge (in addition the age-related variation of adaptive shifts with new target directions approached statistical significance). Thus, both analyses, the analysis of the residuals and the analysis of the subgroups without explicit knowledge, converge on the conclusion that adaptive shifts for practiced target directions reveal an age-related variation beyond that of explicit knowledge.

### VISUAL CLOSED-LOOP TEST

In the final block of visual closed-loop trials, the mean initial direction errors for the young participants were -13.7 and -27.9° for the practiced and new target directions, respectively, and for the older participants the mean initial direction errors were -31.6 and -49.5°. In a two-way ANOVA both the main effects of age group, *F*(1,35) = 18.2, *p* < 0.01, and target set, *F*(1,35) = 30.2, *p* < 0.01, were significant, but not the interaction, *F* < 1. Movement times were 2286 and 2562 ms in the group of young participants for practiced and new targets, respectively, and in the group of older participants they were 3140 and 3373 ms. The difference between the two age groups was statistically significant, *F*(1,35) = 15.7, *p* < 0.01, and so was the difference between practiced and new target directions, *F*(1,35) = 24.5, *p* < 0.01.

## DISCUSSION

The present findings add to a consistent set of observations on age-related changes of adaptation to visuo-motor rotations. These reveal a decline of explicit components of adaptation across the adult age range, but not of implicit components, and they do so with different experimental setups and different types of movements (e.g., McNay and Willingham, 1998; Buch et al., 2003; Bock, 2005; Heuer and Hegele, 2008). For example, Heuer and Hegele (2008) used a monitor in front of the participants on which visual feedback was presented, and participants performed accurate aiming movements during practice. In contrast, Bock (2005) used a mirror arrangement by which visual feedback was presented in the same plane in which the movements were performed, and participants performed rapid out-and-back movements during practice. Thus, the basic pattern of findings is quite robust.

The parallel age-related changes of behavioral adjustments in the presence of the visuo-motor rotation (adaptive shifts) and of explicit knowledge can be taken to suggest that the poorer acquisition of explicit knowledge is the only factor underlying the age-related decline of adaptation to visuo-motor rotations. If this were the case, age-related changes of adaptive shifts should no longer be present if explicit knowledge were the same in the age groups compared. This was indeed what we observed initially (Heuer and Hegele, 2008). However, when explicit knowledge was boosted by way of dedicated practice, we found a persistent age-related variation of adaptive shifts at high levels of explicit knowledge, but not at low levels (Hegele and Heuer, 2013). This finding suggests that older adults make less use of explicit knowledge for strategic movement corrections than young adults do.

In the present study we used a new practice task with a categorical marker of success or failure. This task was intended to enhance the contribution of model-free reinforcement learning to visuo-motor adaptation. With this type of practice we observed persistent age-related changes of adaptive shifts at all levels of explicit knowledge, also when no explicit knowledge was present. Thus, there was a decline of adaptation that was independent of the level of explicit knowledge. Note that explicit knowledge was not boosted as in the study of Hegele and Heuer (2013). Therefore participants, in particular the older ones, acquired no really high levels of explicit knowledge at which particularly strong residual age-related declines of adaptive shifts have been observed.

Consistent with the considerations that led us to use the particular practice task, we claim that the age-related decline of visuo-motor adaptation beyond explicit knowledge results from an age-related decline of reward-based reinforcement learning. This claim relates our present findings, first, to the recent evidence according to which reinforcement learning can contribute to visuo-motor adaptation (see Haith and Krakauer, 2013, for an overview) and, second, to the well-established role of the dopaminergic neurotransmitter system for reinforcement learning (Schultz et al., 1997; Schultz, 1998, 2007) and its age-related changes (e.g., Dreher et al., 2008). In fact, age-related changes of the dopaminergic system have been invoked to account for quite a number of cognitive-aging phenomena (cf. Li and Sikström, 2002).

Reward-based reinforcement learning in the presence of a visuo-motor rotation typically exhibits generalization across a limited range of target directions (e.g., Izawa and Shadmehr, 2011). In the present study, there were five target directions during practice, and three additional new target directions during the tests. Explicit shifts were observed equally for practiced and new targets, confirming previous observations of generalization across directions (Heuer and Hegele, 2008, 2011), but adaptive shifts generalized only partially. In contrast to the practiced target directions, the age-related variation of adaptive shifts for new target directions was fully predictable from the age-related variation of explicit shifts, and there was no reliable residual difference when explicit knowledge in the two age groups was the same. The difference between the residual age-related variations observed for practiced and new target directions is consistent with the claim that it reflects age-related variations of reward-based reinforcement learning. However, limited generalization across target directions is not specific for reinforcement learning but is a more general characteristic of implicit components of adaptation (cf. Heuer and Hegele, 2008). Thus, it does not reliably discriminate between model-free and model-based learning. Nevertheless, if residual age-related variations had been found for new target directions as for the practiced ones, this would have provided evidence against our specific claim about the role of reinforcement learning.

The new findings of the present study were observed against a background of results that confirm previous observations: Both adaptive shifts and explicit shifts were stronger in the young than in the older participants, and explicit shifts were not even statistically significant in the older age group. In contrast, after-effects did not exhibit an age-related variation. Only explicit shifts generalized fully from the practiced to the new target directions, whereas after-effects were absent for new target directions, and for adaptive shifts there was partial transfer. Adaptive shifts, but not after-effects, were positively correlated with explicit shifts. Different from previous studies, for after-effects we observed a small, but significant, negative correlation with explicit shifts. This could be a chance result, but it could also reflect a weak compensatory relation between strategic corrections and implicit adjustments as observed in studies of adaptation to visuo-motor rotation (e.g., Taylor and Ivry, 2011) and prism-adaptation studies (e.g., Redding and Wallace, 1993).

At the end of the present experiment participants were tested in a block of visual closed-loop trials. The motivation for this test was somewhat secondary. Adaptive shifts, after-effects, and explicit shifts serve to assess the characteristics of internal representations of visuo-motor transformations. They are measured under visual open-loop conditions. However, in everyday life, and in working life in particular, the presence of visual feedback is more typical. In the presence of visual feedback, closed-loop control adds to the mastery of visuo-motor transformations. Thus one may ask whether – under such more natural conditions – the quality of internal representations is important for performance at all. To answer this question, we added the visual closed-loop test at the end of the experiment.

As compared with the visual open-loop tests, movement time was much longer in the closed-loop test, indicating the time needed to reach the target accurately under control of visual feedback. In addition the movement-time difference between the two age groups was much stronger than in the open-loop tests, indicating the slower closed-loop processing of older adults. The variations of the initial direction errors, which were measured 200 ms after the start of the movements, reflect the variations of the quality of the internal representations of the transformation and the correct movements. Consistent with the variations of adaptive shifts, they were larger in older than in young adults, and they were larger for the new than for the practiced target directions. The larger initial direction errors of older adults and for new rather than practiced target directions were associated with longer movement times. This pattern of findings suggests that feedback-based corrections need the more time the less accurate open-loop control is, that is, the poorer the accuracy of the internal representations of the visuo-motor transformation. Thus, the observations on internal representations of visuo-motor transformations, as they are made in visual open-loop tests, are reflected in the performance under visual closed-loop conditions and are not fully overridden by feedback-based control.

Finally, we want to comment briefly on the age of our older participants. In the present study, as in our previous studies of age-related variations of visuo-motor adaptation (Heuer and Hegele, 2007, 2008, 2009, 2010, 2011; Hegele and Heuer, 2010a,b,c, 2013), we compared young adults with older adults in the age range of 50-67 years. This contrasts with other studies on age-related variations of visuo-motor adaptation (McNay and Willingham, 1998; Bock and Schneider, 2002; Buch et al., 2003; Bock, 2005; Bock and Girgenrath, 2006; Seidler, 2006), in which the older adults were generally above 60 and up to 80 years of age. There were two reasons for us to study younger older groups. First, we wanted to tap age-related changes that are still relevant for working life. Second, we wanted to identify changes that occur early during the lifespan. In general, motor impairments become progressively severe at older adult age (e.g., Szafran, 1951; Teeken et al., 1996; Yan et al., 1998). Thus the changes that we have identified at older working age, namely the poorer acquisition of explicit knowledge (Heuer and Hegele, 2008), the weaker strategic corrections derived from good explicit knowledge (Hegele and Heuer, 2013), and the reduced model-free reinforcement learning of correct movements as revealed in the present study, may be amplified at age 60 and beyond, and additional types of changes might also accrue. However, after-effects turned out to be quite immune against increasing age thus far, also in studies with older age groups. They are generally considered to reflect implicit model-based learning.

## Conflict of Interest Statement

The authors declare that the research was conducted in the absence of any commercial or financial relationships that could be construed as a potential conflict of interest.
